# Multi-objective design of multi-material truss lattices utilizing graph neural networks

**DOI:** 10.1038/s41598-025-86812-3

**Published:** 2025-01-25

**Authors:** Ramón Frey, Michael R. Tucker, Mohamadreza Afrasiabi, Markus Bambach

**Affiliations:** Advanced Manufacturing Lab, ETH Zürich, Leonhardstrasse 21, 8092 Zurich, Switzerland

**Keywords:** Mechanical engineering, Scientific data, Computational methods

## Abstract

The rapid advancements in additive manufacturing (AM) across different scales and material classes have enabled the creation of architected materials with highly tailored properties. Beyond geometric flexibility, multi-material AM further expands design possibilities by combining materials with distinct characteristics. While machine learning has recently shown great potential for the fast inverse design of lattice structures, its application has largely been limited to single-material systems. In this work, we propose a novel approach that incorporates material properties as edge features within the graph representation of multi-material truss lattices, utilizing graph neural networks (GNNs) to develop a fast and efficient inverse design framework. We validate this framework by designing lattices with tunable thermal expansion and stiffness properties, showcasing its ability to explore a broad and flexible design space. We showcase the framework’s inverse design capabilities for both single and multi-objective optimization tasks and assess its limitations. Additionally, we demonstrate the superior capacity of GNNs in capturing structure-property relationships for multi-material systems. We anticipate that the continued advancement of GNN-assisted inverse design will play a key role in unlocking the full potential of multi-material truss lattices.

## Introduction

Additive Manufacturing (AM) enables not only the fabrication of parts with intricate geometries but also the creation of materials with precisely engineered internal structures, known as architected materials or metamaterials. These materials come in various forms, such as truss-, shell-, and plate-based lattices, which can be manufactured using AM across multiple length scales and material types. Applications span a wide range, including enhanced sound absorption^1^, improved heat transfer^2,3^, vibration control^4^, and even the production of feed spacers for ultra-filtration^5^. Furthermore, the incorporation of multiple materials has been leveraged to broaden the range of achievable properties^6,7^. Truss lattices in particular allow for easy incorporation of multiple materials and have been employed to boost energy absorption^8^ and achieve metamaterials with large negative poisson ratios^9^. Zero thermal expansion (ZTE) lattices, which intrinsically require multiple materials, have also been demonstrated using various truss lattice configurations^10–12^. Despite these advancements, designing complex architected materials remains a significant challenge. The reliance on human intuition and trial-and-error approaches quickly becomes impractical due to the vast number of design variables involved.

**Fig. 1:**
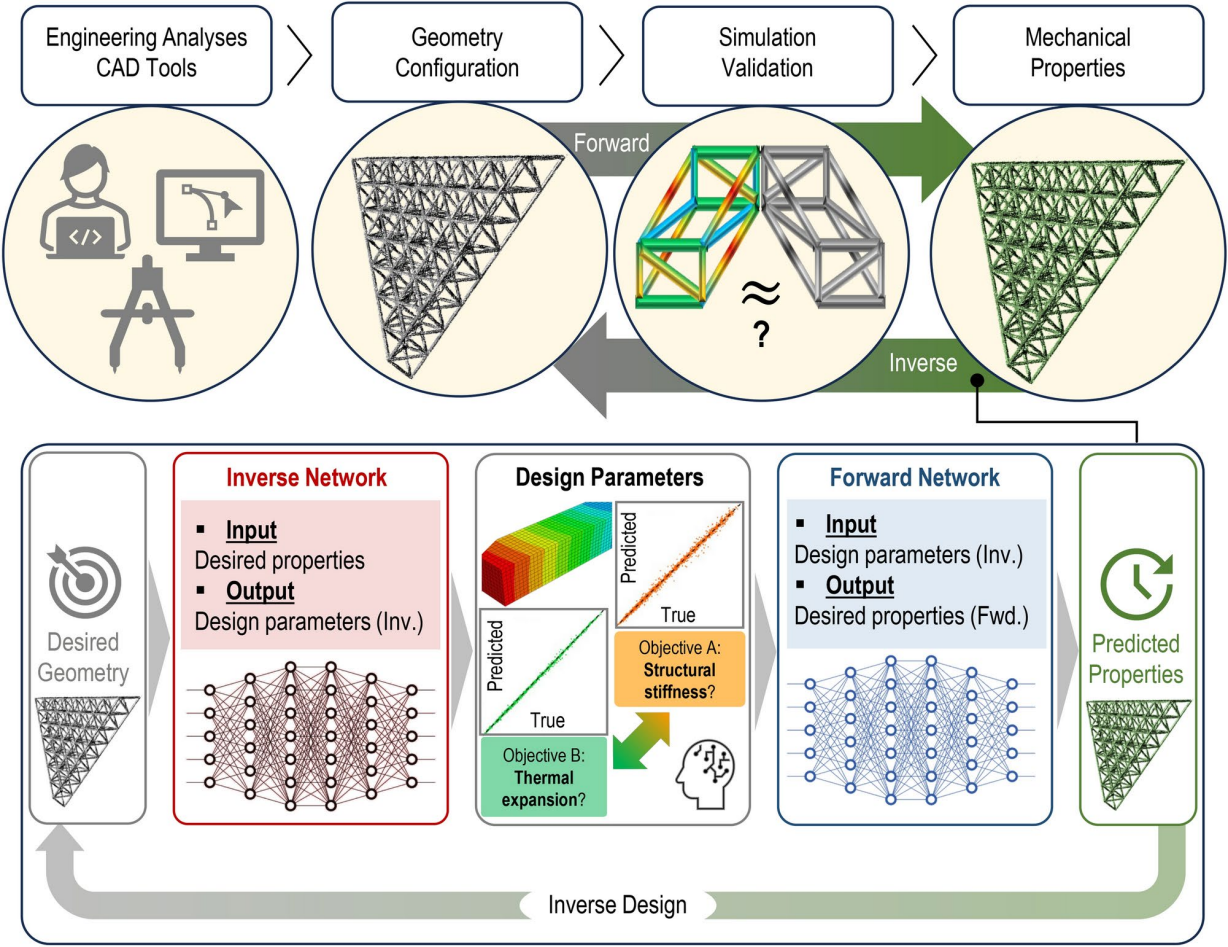
Schematic of ML-based inverse design. Data generated automatically in a conventional forward simulation pipeline can be utilized to train ML models that can directly and quickly output a suitable geometry for a set of desired properties.

Topology optimization, a well-established technique in structural design, has been applied to metamaterial unit cell design for various applications^13,14^. However, it is notoriously computationally demanding. Alternative approaches, such as brute-force search^15^ and the creation of look-up tables via design databases^16^, have also been explored. Yet, as the problem’s dimensionality increases, especially when continuous design variables are involved, these methods become incomplete, often failing to capture extreme designs. Nonetheless, these databases can be leveraged by machine learning (ML) to create fast surrogate models or even direct inverse design models, generating suitable geometries based on desired properties, as illustrated in in Fig. 1.

Various representations, ML models, and algorithmic strategies have been employed to predict the properties and inversely design composites, shell lattices, and truss lattices^17^. Among these, truss lattices stand out for their flexibility, resolution-independent representation, and ease of manufacture for multi-material systems, making them the primary focus of this work. Maurizi et al.^18^ demonstrated that combining simple 2D building blocks can significantly improve buckling resistance. They integrated a fast ML surrogate model with a genetic algorithm to identify lattices with superior buckling strength. However, their study was limited to only two building blocks, and a large portion of the finite design space still had to be computed to train the ML model. Bastek et al.^19^ generated truss lattices by combining selected cubic truss unit cell building blocks, applying affine transformations, and utilizing a direct inverse ML model to design a target anisotropic stiffness tensor. While this approach offers excellent speed and accuracy, it depends heavily on the correct human selection of building blocks and requires that the combination of a few building blocks can cover a sufficiently large property space. Zheng et al.^20^ leveraged the natural graph representation of truss lattices to create a flexible design space, training a Variational Autoencoder (VAE) on it. They then used latent space optimization to design lattices with desired properties, such as high stiffness or minimal Poisson’s ratio, surpassing the limits of the training data. Their work also demonstrated the ability to design structures with specific stress-strain curve shapes.

A key limitation of the approaches discussed above is the lack of consideration for multi-material design. Incorporating multiple materials enables new property combinations and enhances design capabilities but also significantly increases the complexity of the inverse design task due to the added degrees of freedom. Consequently, most existing work on ML-based multi-material truss lattice design focuses on adjusting parameters^21^ or altering materials^22,23^ in a limited set of fixed topologies. This may be due to the difficulty in accurately describing and predicting the behavior of complex multi-material truss networks using basic vector representations and fully connected neural networks (FCNNs).

Graph neural networks (GNNs) extend convolutional neural networks (CNNs) to graph-structured data, making them applicable in a variety of domains such as computer vision^24^, social network recommendations^25^, and drug side effect prediction^26^. GNNs are also increasingly used in mechanics^27^. In the context of architected materials, GNNs have been employed to train surrogate models for predicting compressibility^28^, compression strength^29^, and deformation mechanisms^30^ of truss lattices. Meyer et al.^31^ introduced a graph representation for shell lattices and combined a GNN surrogate model with a genetic algorithm to optimize structures for desired anisotropic stiffness and thermal conductivity. However, GNNs’ potential to incorporate edge features—such as truss thickness, material properties, or curvature—has not yet been fully explored.

The aim of this study is therefore to answer the following research questions:

- Can the previously reported graph-representation-based VAE^20^ be extended to handle multi-material truss lattices?
- Can GNNs improve the modeling of structure-property relationships in multi-material truss lattices by incorporating material assignments as edge features?
- Can this framework be used for inverse design, including multi-objective design goals, and can it generate new designs that outperform those in the training data?

The remainder of this paper is structured as follows. The Methods section begins by outlining the parameterization, dataset generation, and finite element modeling of the multi-material truss lattices. Next, we describe the machine learning model, focusing on the GNN convolution layers, training process, and optimization procedures. In the Results and Discussion section, we first assess the validity of the design space, and then compare the performance of GNNs and FCNNs in predicting structure-property relationships for both single and multi-material lattices. Finally, we evaluate the model’s inverse design capabilities by optimizing for maximal negative thermal expansion and for a combination of zero thermal expansion (ZTE) and maximal stiffness. The paper concludes with a summary of the approach’s strengths and limitations, along with suggestions for future work.

## Methods

### Representation and dataset generation

**Fig. 2:**
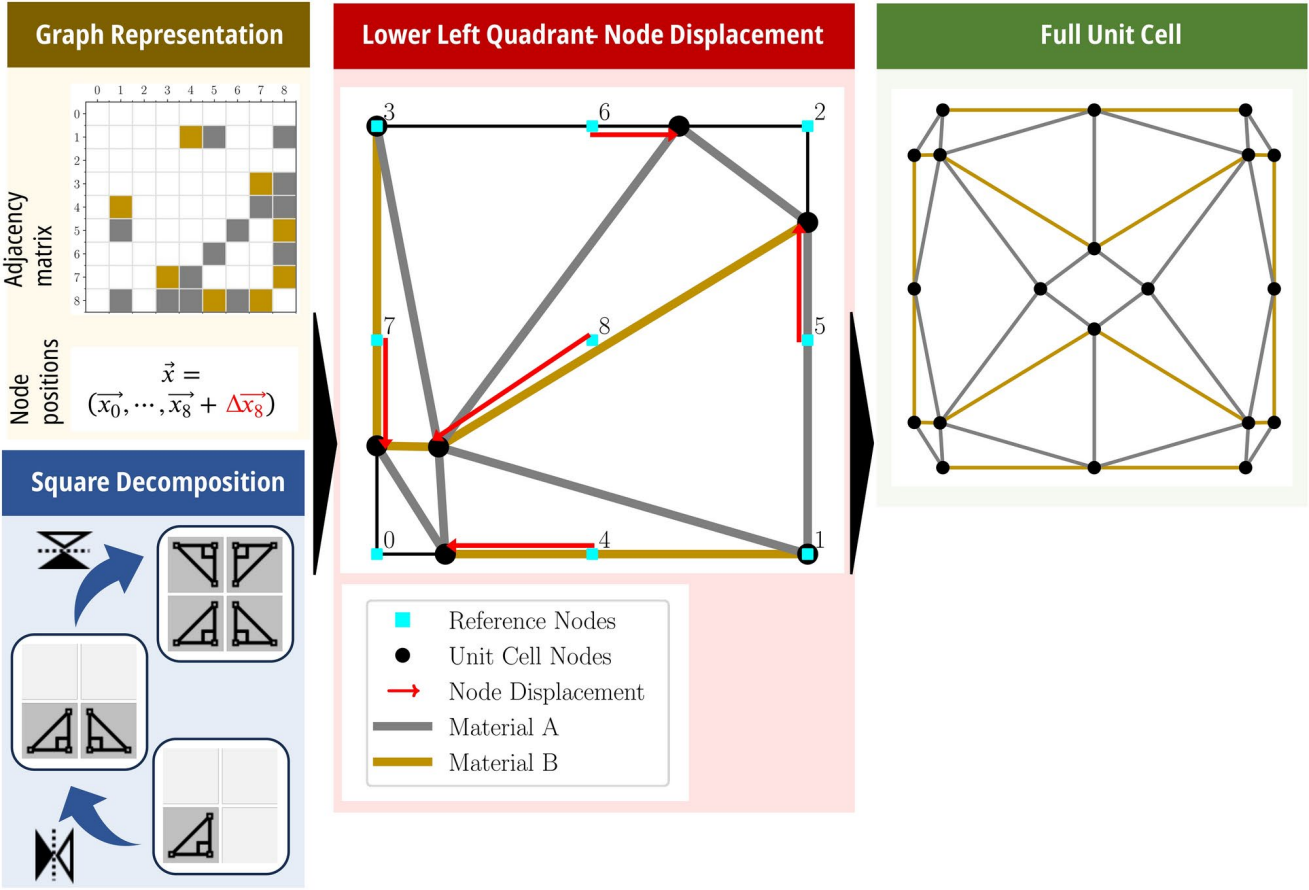
Graph representation of orthotropic unit cells for multiple materials. The node coordinates and connections, encoded with their material in the adjacency matrix, are defined in the lower left quadrant of a square. By mirroring twice, the full unit cell is retrieved.

A well-defined design space should balance the objectives of maximal generality and minimal representation complexity. While a graph representation of truss lattices (and GNNs) can theoretically handle an arbitrary number of unordered nodes, an inverse design framework requires a finite set of degrees of freedom, and imposing order helps with comparing output structures (e.g., in the loss function of an ML model). Inspired by cube decomposition^32^, we adopt a 2D square decomposition approach, dividing a square with two orthogonal symmetry planes. This reduces the degrees of freedom while ensuring tiled connectivity. One-quarter of the square contains nodes at each corner, along the edges, and at the center, with their positions described by displacement vectors. These nodes can be connected in any configuration, including leaving some nodes unconnected, and each connection is made of either material **A** or material **B**. The node coordinates, described by the initial position plus displacements, form the node features of the graph $G$. The adjacency matrix encodes the connectivity of $G$, where 0 indicates no connection, and 1 and 2 indicate connections made of material **A** or **B**, respectively. A schematic of the unit cell generation and representation is shown in in Fig. 2.

A dataset of unit cells was created using the following 5-step procedure for each sample:

1. For each unit cell, generate a displacement vector $\Delta \textbf{x} = (\Delta x_1, \ldots , \Delta x_6)$, where each component $x_i$ is drawn from a uniform distribution:
  $$x_i \sim \mathscr {U}(-0.4, 0.4)$$
  This limited range prevents extremely short connections that would violate the assumption of beam-like behavior.
2. Randomly select $n$ nodes, where $n$ is an integer drawn from the discrete uniform distribution:
  $$n \sim \mathscr {U}_{\mathbb {Z}}(3, 9)$$
3. For the set of $n$ nodes, perform a Delaunay triangulation to establish initial connections between the nodes. This creates a set of connections $\mathscr {C}_\text {initial}$, ensuring all nodes are connected without intersections.
4. Randomly remove some connections from $\mathscr {C}_\text {initial}$ to create the final set of connections $\mathscr {C}_\text {final}$, while maintaining the overall connectivity of the graph.
5. Randomly assign material **A** or **B** to each connection in $\mathscr {C}_\text {final}$.

The full unit cell is then constructed for each sample, and the corresponding properties are calculated as described in the following section. The dataset size was chosen to be 300k samples to strike a balance between computational cost and model performance.

### Finite element modeling

For each unit cell, a constant beam width is assumed, which is adjusted to maintain a uniform density of 0.15. The materials considered are the steels 316L and 17-4PH, with coefficients of thermal expansion of 12.6 × 10^−6^ and 16.1 × 10^−6^, respectively. The thermal expansion and effective stiffness tensors of the unit cells are calculated independently, meaning that thermally induced stresses are not considered when calculating the stiffness tensor.

To compute the homogenized macroscopic stiffness tensor, we adopt the method of Vigliotti et al.^33^, using rectangular cross-section Timoshenko-beam elements. The thermal expansion coefficient is calculated assuming linear elastic material behavior, constant CTE values for the constituent materials, and axial-only thermal expansion. Periodic boundary conditions are applied to approximate the thermal expansion behavior of each unit cell. Both property calculations were implemented in C++ using the ae108 library^34^.

### ML models

**Fig. 3:**
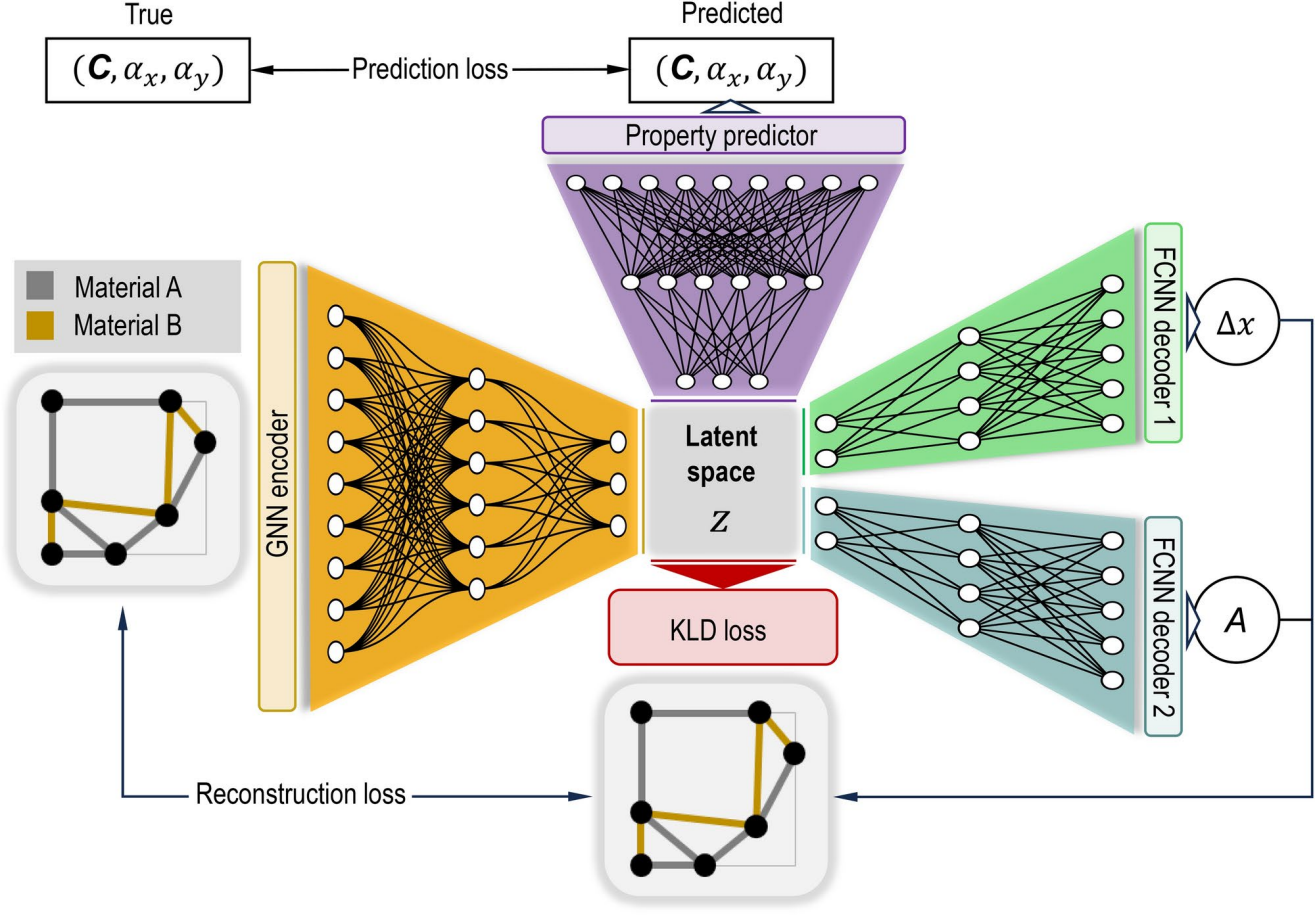
Schematic of the ML model architecture. A GNN encoder and two FCNN decoders make up the VAE. A property predictor is jointly trained to predict the stiffness tensor and thermal expansion vector based on the latent space representation.

The ML framework comprises a VAE with a GNN encoder, two FCNN decoders, and an FCNN property predictor, as illustrated in in Fig. 3. The input to the VAE is a graph representation of the lower-left quadrant of a full truss lattice unit cell, with node positions encoded as node features. The input graph is fully connected, with one-hot encoded edge features representing “no connection,“connection with material **A**,” or “connection with material **B**.”

In the encoder, the node and edge feature vectors $\textbf{h}_i$ and $\textbf{e}_{ij}$ are projected into a higher-dimensional latent space followed by graph convolution layers. The convolutions operate on the concatenated node and edge features, with the updated node features, $\textbf{h}_i^{(k+1)}$, computed as:

1 $$\begin{aligned} \textbf{h}_i^{(k+1)}&= \textbf{h}_i^{(k)} \bigoplus _{j \in \mathscr {N}(i)} \psi \left( \textbf{h}_i^{(k)}, \textbf{h}_j^{(k)}, \textbf{e}_{ij} \right) \text {with} \end{aligned}$$

2 $$\begin{aligned} \quad \psi \left( \textbf{h}_i^{(k)}, \textbf{h}_j^{(k)}, \textbf{e}_{ij} \right)&= \sigma \left( \textbf{W}_s^{(k)} \cdot \sigma \left( \textbf{W}_f^{(k)} \cdot \left[ \textbf{h}_i^{(k)} \Vert \textbf{h}_j^{(k)} \Vert \textbf{e}_{ij}\right] + \textbf{b}_f^{(k)}\right) + \textbf{b}_s^{(k)}\right) \end{aligned}$$

where $\textbf{W}_f^{(k)}$, $\textbf{W}_s^{(k)}$ and $\textbf{b}_f^{(k)}$, $\textbf{b}_s^{(k)}$ are the learnable weight matrices and bias vectors for layer *k*, $\sigma$ is the activation function (Exponential Linear Unit, ELU), $\Vert$ denotes concatenation, and $\bigoplus$ represents the aggregation operator (e.g., summation or maximization). After the final convolutional layer, global mean pooling is applied, and the output is projected into the latent space dimension through a linear layer.

The decoder comprises two separate FCNNs: one for reconstructing node displacements and the other for generating the adjacency matrix. The property predictor is an FCNN that takes a latent space vector as input and outputs the four independent components of the orthotropic stiffness tensor, along with the thermal expansion vector. The VAE-models with the FCNN encoder used for comparison follows the same structure as reported by Zheng et al.^20^, with slight modifications to the hyperparameters. For an overview of all model parameters see Supplementary Tables S1 and S2.

#### Model training

Due to the imbalanced nature of the dataset, which contains few examples of extreme thermal expansion values, samples with thermal expansion outside the interval [− 10 × 10^−6^ K^−1^, 35 × 10^−6^ K^−1^] were discarded from the dataset. Given the translational invariance of the full lattice structures, data augmentation is possible by considering each quadrant of the unit cell as representative of the full lattice. This approach resulted in a final dataset of 1,197,704 entries, of which 90% were used for training and 5% were set aside for validation and testing each. Min–max scaling was applied, scaling each component of the stiffness tensor to the range [0, 1] and thermal expansion values to the range [− 1, 1].

The VAE and the property predictor are jointly trained using the following combined loss function:

3 $$\begin{aligned} \mathscr {L} = \overbrace{\frac{1}{N} \sum _{i=1}^{N} ||\Delta x_i - \hat{\Delta x_i}||^2 - \sum _{i=1}^{N} \sum _{c=0}^{2} A_{i,c} \log (\hat{A}_{i,c})}^{\text {Reconstruction Loss}} + \overbrace{\sum _{i=1}^{N} | y_i - \hat{y_i} |}^{\text {Property Loss}} + \overbrace{\frac{1}{2} \sum _{i=1}^{N} \sum _{j=1}^{d} \left( \sigma _{i,j}^2 + \mu _{i,j}^2 - 1 - \log \sigma _{i,j}^2 \right) }^{\text {Kullback-Leibler Divergence}} \end{aligned}$$

The reconstruction loss ensures that the decoded structures closely match the original ones, both in terms of node displacements and the adjacency matrix. For the continuous node displacements, a mean squared error (MSE) loss is employed, while a cross-entropy loss is applied to the categorical adjacency matrix. The property prediction loss uses a weighted $\mathscr {L}_1$ loss to ensure accurate predictions for both stiffness and CTE. Lastly, the Kullback-Leibler divergence (KLD) encourages the probability distribution of the latent space to conform to a standard Gaussian distribution.

#### Gradient-based optimization

The continuous latent space, along with the property predictor, can be utilized for gradient-based optimization. However, due to the discrete nature of truss lattices and the potential for generating invalid structures (such as those with intersections or disconnected components), direct optimization in the latent space is not recommended. Instead, during each optimization step, the latent space vector is first decoded and then re-encoded by the VAE to ensure that the output remains valid.

To facilitate smooth optimization, the discreteness of the edge feature is relaxed by applying a softmax layer to the decoder’s output. Additionally, to enhance the diversity of the optimization results, stochasticity can be introduced by replacing the softmax layer with a Gumbel-softmax and adjusting its temperature parameter.

## Results and discussion

### Design space

A fundamental requirement for data-driven inverse design is the existence of a vast design space, along with a dataset that adequately samples this space. For the application under consideration, the design space must encompass a wide range of CTE and stiffness values.

**Fig. 4:**
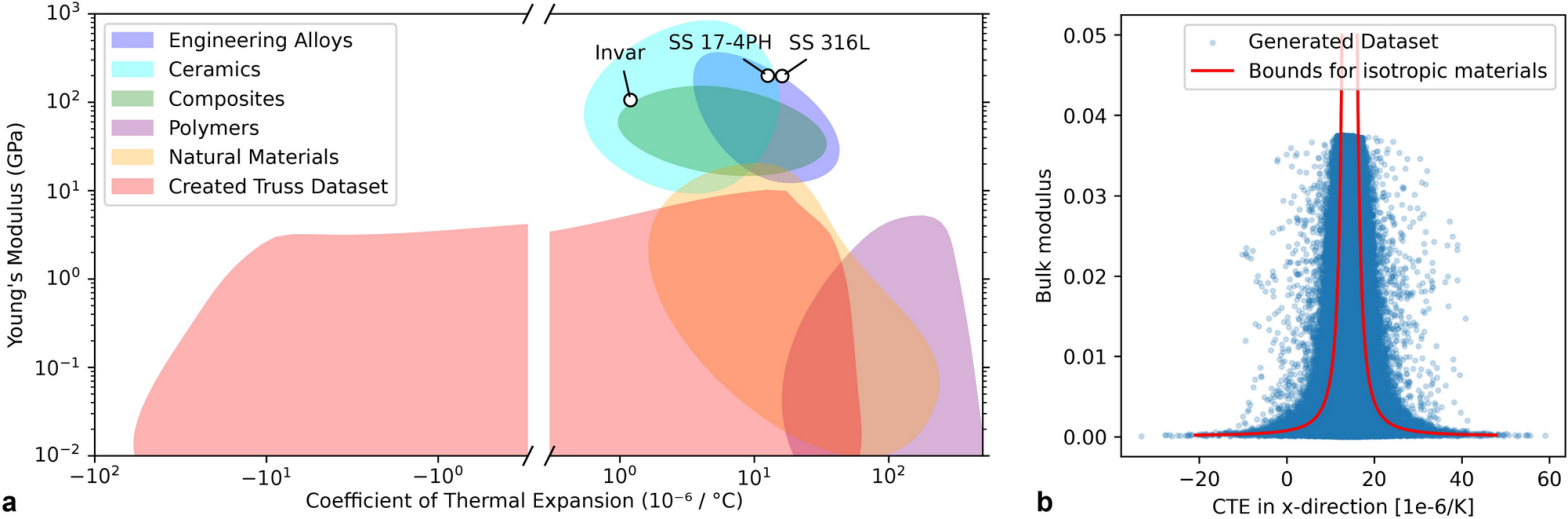
Property space of dataset. (**a**) Map of Youngs modulus versus CTE, showing conventional materials^36^ and the property space covered by the dataset. (**b**) CTE$_x$ vs bulk modulus of the structures in the dataset. The red line shows the theoretical bounds for isotropic materials, which the orthotropic unit cells can exceed in a single direction.

The unit cells included in the created dataset indeed fulfill this requirement and even surpass the limits of conventional materials, as illustrated in the Young’s modulus versus CTE map shown in Fig. 4a. This is achieved despite the relatively low difference in CTE between the constituent materials, particularly when compared to other negative thermal expansion (NTE) lattice designs that often rely on material combinations with large CTE differences, such as Al-Ti^10^ or steel-invar^11^, which are less compatible with multi-material AM processes.

It is noteworthy that stiffness and thermal expansion are not independent properties; rather, there exists an intrinsic trade-off between them. Extremal values of thermal expansion can typically only be achieved at the expense of stiffness. For isotropic composites, theoretical bounds for the CTE and bulk modulus can be derived^35^. However, due to the orthotropic symmetry of the unit cells being considered, thermal expansion in one direction can be compensated by expansion in another direction. This compensation allows the unidirectional CTE values to exceed the theoretical bounds established for isotropic cases, as depicted in Fig. 4b. This further reinforces the appropriateness of the selected design space and the dataset generation method.

### GNN versus FCNN encoder

**Fig. 5:**
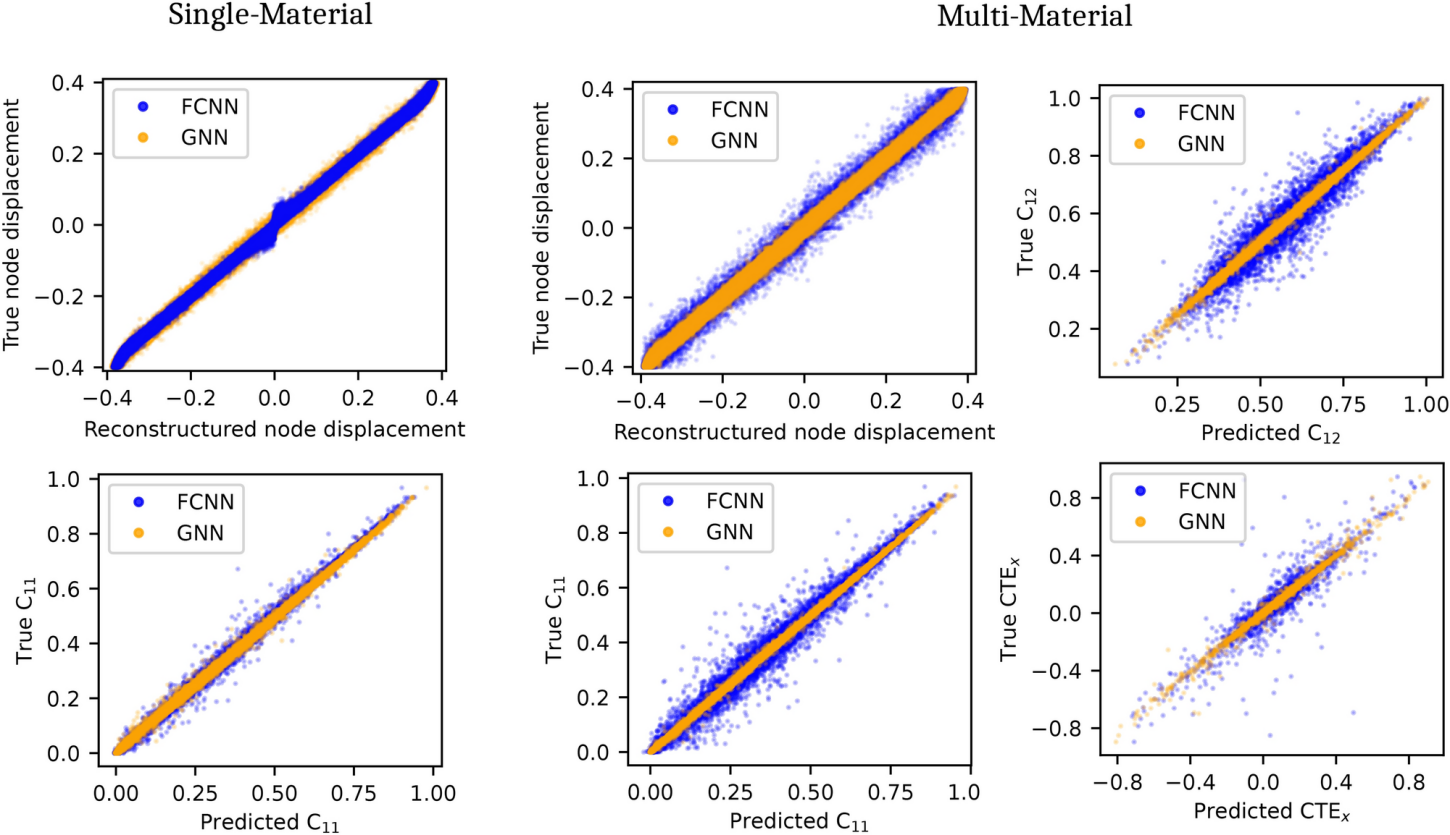
Performance comparison of GNN and FCNN encoder on test set. Nearly equivalent performance is observed for single-material lattices, but the GNN model outperforms the FCNN significantly for the multi-material case.

To establish a performance baseline, the GNN- and FCNN-VAE were trained on a single-material dataset, predicting only the four components of the orthotropic stiffness tensor. As shown in Fig. 5, the FCNN model achieved high reconstruction accuracy for the node positions ($\hbox {R}^2$> 0.999) and connectivity (accuracy > 0.999), along with accurate stiffness tensor predictions (mean $\hbox {R}^2$ > 0.993). The GNN model demonstrated comparable performance, with slightly lower reconstruction accuracy ($\hbox {R}^2$> 0.998 and accuracy > 0.999) but higher accuracy for stiffness predictions (mean $\hbox {R}^2$ > 0.996).

For multi-material unit cells, the models predicted both the stiffness tensor and the thermal expansion coefficients. As shown in Fig. 5, the performance of the FCNN model decreased in terms of reconstruction accuracy ($\hbox {R}^2$ = 0.998 and accuracy 0.989) and stiffness prediction (mean $\hbox {R}^2$ = 0.970). Additionally, the CTE prediction exhibited extreme outliers, resulting in a mean $\hbox {R}^2$ of only 0.859. In contrast, the GNN encoder model maintained excellent performance for the multi-material case, achieving high reconstruction accuracy ($\hbox {R}^2$ = 0.999 and accuracy > 0.999) and reliable stiffness prediction (mean $\hbox {R}^2$ = 0.998). It also provided good accuracy for CTE predictions (mean $\hbox {R}^2$ = 0.987). Results for all stiffness tensor and CTE components are shown in Supplementary Fig. S1.

**Table 1:** Summary of performance metrics for FCNN and GNN encoders in unit cell reconstruction and prediction of stiffness and CTE.

Model	# Materials	Reconstruction	Connectivity	Stiffness prediction	CTE prediction
FCNN-VAE	1	> 0.999	> 0.999	> 0.993	–
GNN-VAE	1	> 0.998	> 0.999	> 0.996	–
FCNN-VAE	2	0.998	0.989	0.970	0.859
GNN-VAE	2	0.999	> 0.999	0.998	0.987

These results, summarized in Table 1, demonstrate the advantages that GNNs offer over FCNNs when handling graph-structured data that includes edge features. The incorporation of GNNs successfully extended the VAE model^20^ to multi-material lattices. This superior performance of GNNs is anticipated to apply to other scenarios, such as lattices with curved beams or varying beam shapes and thicknesses, which can be represented through continuous edge features.

### Optimization in latent space

#### Single objective: negative thermal expansion

**Fig. 6:**
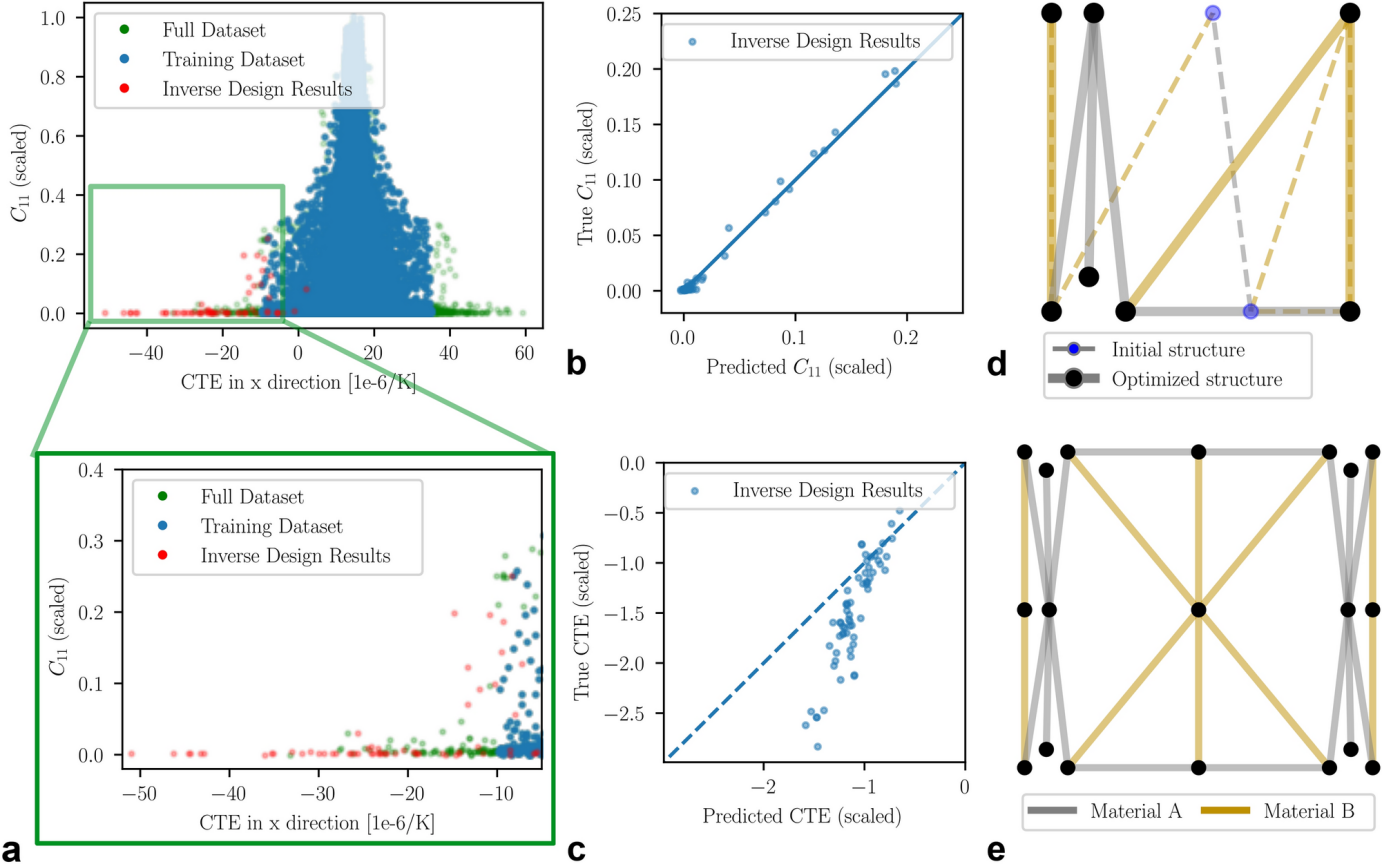
Inverse design results for maximizing NTE. (**a**) CTE vs stiffness in the $x-$direction of the training dataset, the full dataset, and the inverse design results. Most optimized structures reach lower CTE than the training dataset and the best few also exceed the full dataset. (**b,c**) ML prediction versus true values for $\hbox {C}_{11}$ and CTE$_x$ respectively. For the optimized structures, $\hbox {C}_{11}$ is still predicted accurately, while the CTE prediction is quantitatively not accurate but qualitatively still correct. (**d,e**) Changes during the optimization of the best result and the full unit cell of the best result.

Gradient-based optimization was employed to assess the inverse design capability of the model and to identify unit cells with maximally negative CTE values. The optimization began with the 100 unit cells exhibiting the lowest CTE values in the training set, aiming to minimize CTE without imposing any additional constraints. The optimized structures demonstrated significantly lower CTE values compared to the initial structures in the dataset (see Fig. 6a), highlighting the model’s inverse design capabilities. However, the unit cells with the lowest CTE values were excluded from the dataset to enhance the accuracy of the ML model. Thus, a fair comparison between the proposed inverse design approach and a simple lookup table must consider the full dataset.

As shown in the zoom-in of Fig. 6a, most of the inversely designed structures did not exceed the minimum CTE of the full dataset, which is − 33.2 $\times$ 10$^{-6}$ $\hbox {K}^{-1}$. Nevertheless, a few optimized unit cells perform even better, achieving values as low as − 51.1 $\times$ 10$^{-6}$ $\hbox {K}^{-1}$, demonstrating the effectiveness of the proposed approach. It is important to note that while the stiffness predictions remain accurate for the optimized structures (see Fig. 6b), the CTE predictions significantly deviated from the actual values (see Fig. 6c). This discrepancy arises because the model was not trained on such low CTE values, leading to a lack of quantitative generalization. The problem could be mitigated by training the model on the full dataset, including sparse examples with extreme properties, but at the cost of reduced general accuracy. Also, due to the limited extrapolation capabilities of neural networks, the inaccuracy outside the range of values present in the dataset would ultimately remain. Despite the lack of quantitative accuracy, the qualitative trends still hold, which enables the model to find unit cells with extreme properties outside of the dataset property space, as summarized in Table 2.

Figure 6d illustrates the changes made between the initial and the optimized structure, including material modifications, optimized node positions, and the addition of a new connection. This showcases the optimization procedure’s capability to significantly alter the starting structures to enhance their properties. The material change resulted in a triangular configuration within the full unit cell (see Fig. 6e), featuring low-CTE legs and a high-CTE base, a characteristic of known NTE structures. The addition of what may seem like an extraneous connection is a consequence of the imposed fixed density, which reduces the beam widths in the unit cell, facilitating deformation and ultimately leading to a lower CTE. Although this artifact is undesirable, it can be easily addressed through post-processing.

#### Multiple objectives: ZTE and stiffness

Another design case involves optimizing for ZTE while maximizing stiffness, a combination critical for applications such as space-borne optics, which must maintain precise positioning despite significant temperature fluctuations and endure the shocks and accelerations of launch. This multi-objective optimization problem presents a particular challenge because, as previously discussed, low CTE and high stiffness are inherently competing properties. To assess the framework’s capability in addressing this task, an optimization target was established with a CTE of 0 and a $\hbox {C}_{11}$ value exceeding that of the best candidate in the dataset. Optimization was then performed using the 100 best candidates from the dataset, guided by a weighted MSE loss function.

**Fig. 7:**
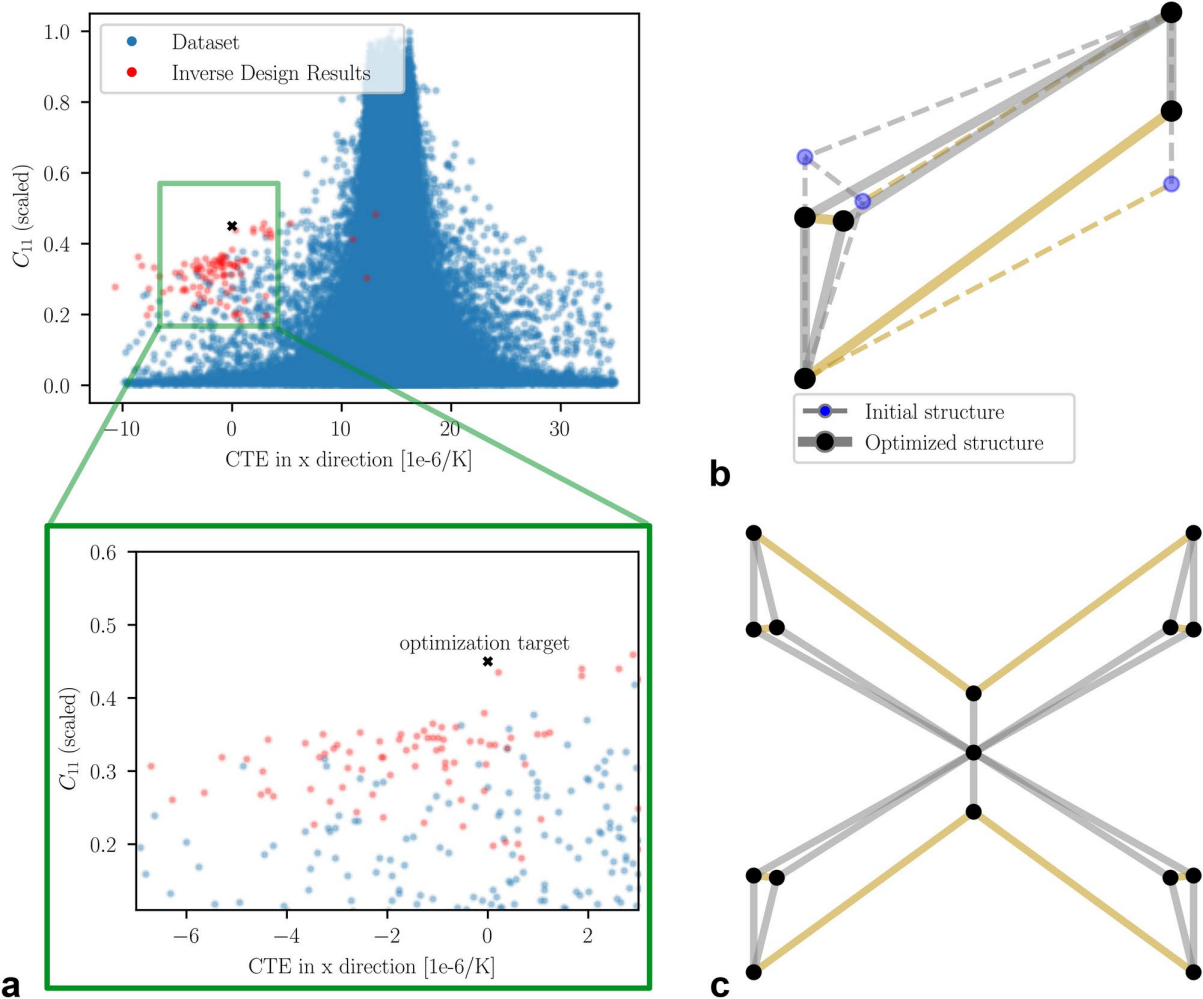
Inverse design results for ZTE and maximal stiffness. (**a**) CTE$_x$ vs $\hbox {C}_{11}$ for the dataset and the inverse design results. Inverse design results cluster around ZTE but with a lot of variation and only one candidate gets close to the optimization goal. (**b,c**) Changes from initial to optimized unit cell quadrant and the full unit cell of the best result.

The distribution of the optimization results is presented in Fig. 7a, revealing mixed success. While the majority of the structures cluster around zero, there is a slight bias towards more negative values. However, their stiffness levels barely surpass those of the best candidates in the dataset. This limitation may stem from the optimization process becoming trapped in a local minimum of the initial topology or from conflicting gradients arising from the two competing optimization objectives. Additionally, the range of values is quite broad, with outliers extending to extreme values <-10 and >10, indicating that the precision of the CTE predictions may be insufficient. This discrepancy could be attributed to the continuous nature of the edge features during optimization, as opposed to the categorical values used during training.

Nonetheless, one structure comes remarkably close to the optimization target, indicated by the black cross in Fig. 7a, and outperforms the best match from the dataset (see Table 2). The number and quality of such positive outliers can vary between different optimization runs. However, due to the speed of the proposed method (100 candidates in 30 seconds), multiple optimization iterations can be performed, potentially yielding promising candidates.

The starting and optimized structure of the best candidate are displayed in Fig. 7b. While the connectivity remains unchanged, two beams have switched materials, and the node positions have been adjusted slightly. The full unit cell is shown in Fig. 7c, revealing that the angle between two of the struts is unreasonably small. This configuration could lead to significant overlap of the beams during manufacturing, which might drastically alter the resulting properties. Such structures should therefore be filtered out or additional solid elements FEM calculations need to be performed that take the overlap into account.

**Table 2:** Overview of the performance improvement of the optimized structures compared to the best candidates in the training and full dataset.

Optimization Target	Best in training dataset	Best in full dataset	Best optimized	Improvement w.r.t training dataset (%)	Improvement w.r.t full dataset (%)
Minimize CTE	− 10	− 33	− 51	230	58
Maximize $\hbox {C}_{11}$ (at ZTE)	0.0045	0.0045	0.0054	19	19

## Conclusion

We developed a GNN-driven framework for inverse design of multi-material truss lattices, based on a versatile design space and data generation process, capable of exploring a vast property space, including extreme CTE values. The truss structures are effectively represented using a graph-based approach, where material properties are encoded as edge features. The subsequent training of a VAE and property predictor demonstrated that the GNN variant significantly outperformed its FCNN counterpart. This highlights the potential of GNNs in capturing the complex structure-property relationships in truss lattices with edge features, including variations in thickness, shape, and curvature.

Through gradient-based optimization in the latent space, the framework successfully realized inverse design for both single-objective (maximizing negative thermal expansion, NTE) and multi-objective scenarios (achieving zero thermal expansion, ZTE, while maximizing stiffness). The optimized designs produced properties that extend beyond the initial training property space. However, it is important to note that the quantitative accuracy of the ML model decreases outside the training domain and that some designs exhibit unreasonably small angles.

To address the problem of unreasonable designs, future work should explore the use of solid element FEM simulations and whether they enable the ML model to learn the effect of overlapping beams implicitly. To increase model performance in extremal property regions, targeted data generation or active learning strategies should be explored. Finally, other application cases that could benefit from incorporating multiple materials should be explored, such as lattices with combined mechanical and thermal conductivity requirements or lattice structures for vibration damping. By doing so, the potential of this approach for designing advanced multi-material truss lattices can be fully realized.

## Supplementary Information

Supplementary Information.

## Data Availability

The raw/processed data required to reproduce these findings cannot be publicly shared at this time as it is part of an ongoing research project, but is available from the corresponding author on reasonable request.
