# Supplementary material for: Multi-objective design of multi-material truss lattices utilizing graph neural networks
Source: Sci Rep. 2025 Jan 25;15:3187. doi: 10.1038/s41598-025-86812-3 (PMC11763062; doi:10.1038/s41598-025-86812-3)
Supplement: Supplementary file 1 — Supplementary Information. [file 41598_2025_86812_MOESM1_ESM.pdf]

Supporting information for

Multi-objective Design of Multi-material Truss Lattices utilizing Graph Neural Networks

Ramón Frey, Michael R. Tucker, Mohamadreza Afrasiabi, Markus Bambach

ML Model Hyperparameters

Model parameters and dimensions of the GNN-VAE and the FCNN-VAE used for comparison are given in Table S1. The details of the GNN-encoder used in the GNN-VAE are provided in Table S2.

	**Encoder**		**Decoder**		**Property Predictor**
	Connectivity A	Node positions x	Connectivity A	Node positions x	
**FCNN-VAE**					
Input dimensions	108 (36)	6	26	20	28
Hidden dimensions	640,640,640,512	256,256,256,128	512,640,640,640	256,256,256,128	512,1024,512,512,256
Output dimensions	26	20	108 (36)	6	6 (4)
Activation functions	ELU		ELU		LeakyReLU
**GNN-VAE**					
Input dimensions	-		28	28	28
Hidden dimensions	-		320,640,640,320	160,320,320,160	512,512,512,512
Output dimensions	-		108 (36)	6	6 (4)
Activation functions	-		ELU		LeakyReLU

Table S1. Model Parameters Overview of the model parameters of the FCNN-VAE and GNN-VAE. Brackets indicate values for the single-material case.

GNN-encoder

	Input dimension node features	Input dimension edge features	GNN-layer node dimension	GNN-layer edge dimension	# of layers
multi-material	2	3	256	128	5
single-material	2	1	128	1	5

Table S2. GNN-encoder parameters

ML Model Performance

The performance comparison of the FCNN-VAE and GNN-VAE is evaluated on a previously unseen test set comprising of 5% of the total dataset. Figure S1 shows the results for the components of the stiffness and CTE tensor that are not shown in the main text (Figure 5).

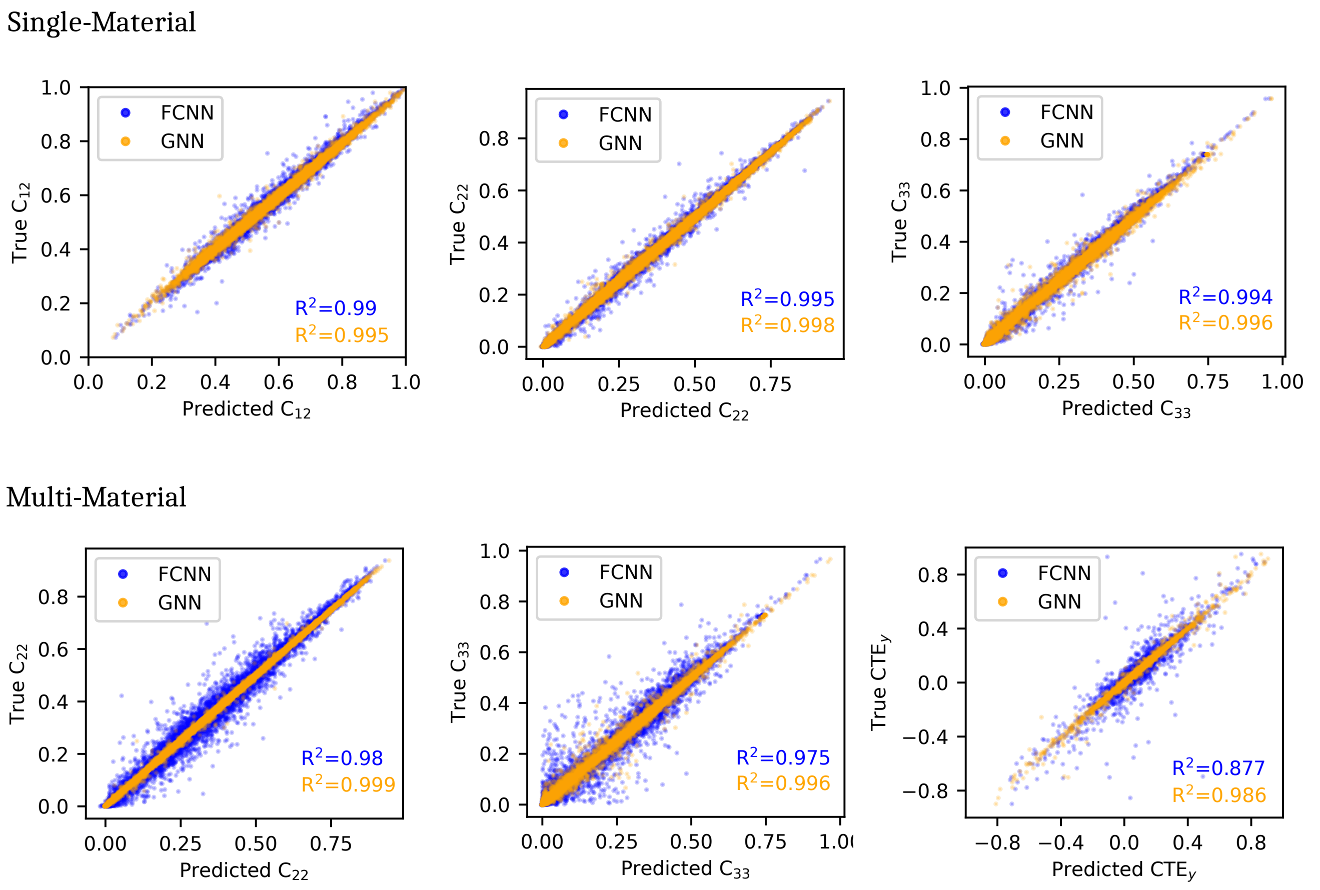

Figure S1. FCNN vs GNN performance comparison Predicted vs true values for the components of the stiffness tensor and CTE vector that are not shown in the main text.
